# High-Dose Radiation as a Dramatic, Immunological Primer in Locally Advanced Melanoma

**DOI:** 10.7759/cureus.417

**Published:** 2015-12-18

**Authors:** Majid Mohiuddin, Harold Park, Sigrun Hallmeyer, Jon Richards

**Affiliations:** 1 Radiation Oncology, Advocate Lutheran General Hospital; 2 Medical Oncology, Advocate Lutheran General Hospital

**Keywords:** immunotherapy, spatially fractionated radiation therapy (grid), metastatic melanoma, ipilimumab, pembrolizumab

## Abstract

A 53-year-old white male presented with a right axillary melanoma that became widely metastatic and progressive despite multiple systemic treatments. He became refractory to ipilimumab (Yervoy) and pembrolizumab (Keytruda). He presented with a very large, painful left posterior neck mass that was 18 x 15 x 8 cm in size clinically. He was treated with a single fraction of 20 Gy using parallel opposed, spatially fractionated GRID radiation therapy (SFGRT), along with concurrent pembrolizumab. He also received 50 Gy in 25 fractions of conventional radiation. After five months of concurrent treatment, the refractory neck mass had completely resolved and he had no lasting side effects. Our dramatic case confirms the synergistic effect of high-dose GRID radiation as a primer for renewed, enhanced immunological response, and we have used this approach successfully on a number of similar patients with rapid and durable results.

## Introduction

The current treatment management for advanced melanoma attempts to enhance the patient’s own immune system to attack evasive cancer cells. Previously, this was done with cytokines, like interleukin-2, to give a general boost at significant toxicity, but recently, considerable progress has been made with the approval of novel immune checkpoint inhibitors in the form of specific human monoclonal antibodies. These newer drugs, such as ipilimumab and pembrolizumab, target and inhibit proteins on T-cells, such as CTLA-4 or PD-1, respectively. By removing a suppressive checkpoint on the immune response, the immune response is enhanced against the cancer cells. Pembrolizumab has shown increased progression-free survival as compared to chemotherapy in ipilimumab-refractory patients, but unfortunately, the response is only seen in a subset of patients [1]. When patients are ineligible for these treatments due to the tumor’s genetic signature, or later prove refractory or unresponsive to these kinds of immunotherapies, they have few options left. We report on a pembrolizumab-refractory patient with an enlarging disease who was dramatically re-sensitized to the same drug by the administration of high-dose radiation.

## Case presentation

Informed patient consent was obtained. No identifying patient information was disclosed in this paper.

A 53-year-old white male originally presented with a Stage IIIA right shoulder and right axillary melanoma and underwent wide local excision and lymph node dissection in August 2011. Two years later, he developed a painful retroperitoneal disease. In April 2013, he underwent surgical resection of the spleen, right adrenal gland, bowel, and kidney that proved he had metastatic melanoma, BRAF-negative.

In June 2013, he started ipilimumab (Yervoy) due to a new, large posterior left neck mass and disease in the retroperitoneum. By August 2013, the ipilimumab was discontinued after four cycles due to dermatitis requiring steroids. The neck mass and retroperitoneal disease responded partially at first, but then grew back by October 2013. 

By January 2014, the neck mass demonstrated 15% further progression. Therefore, the patient was started on high-dose interleukin-2 (IL-2) for two cycles, but he still had progression. He was started on the anti-PD-1 directed monoclonal antibody, pembrolizumab (Keytruda), for five cycles from June to September 2014 but still showed a significant progression of an additional 30% by RECIST (response evaluation criteria in solid tumors) criteria.

**Figure 1:**
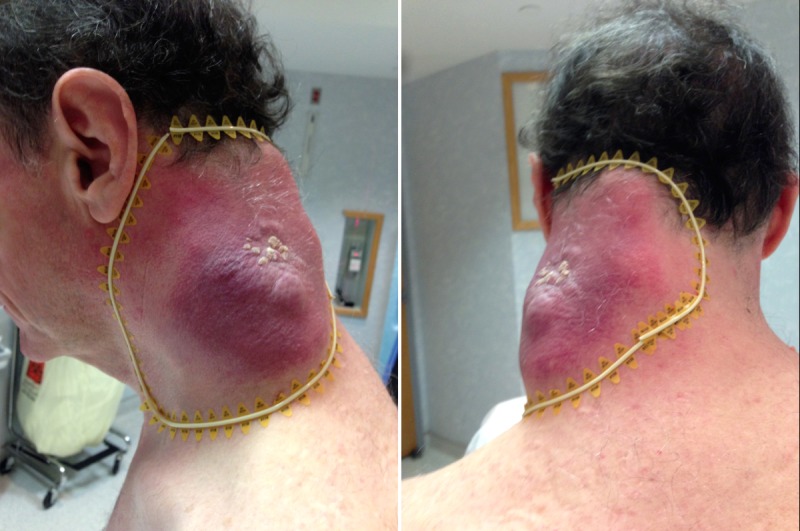
Initial Tumor on Neck The initial tumor on the left posterior neck of 18 x 15 x 8 cm size, demarcated by wire.

In September 2014, he presented with a left posterior neck mass that was 18 cm in the anterior-posterior dimension, 15 cm superior-inferior, and 8 cm thick causing pain and a decreased range of motion (Figure 1).

**Figure 2:**
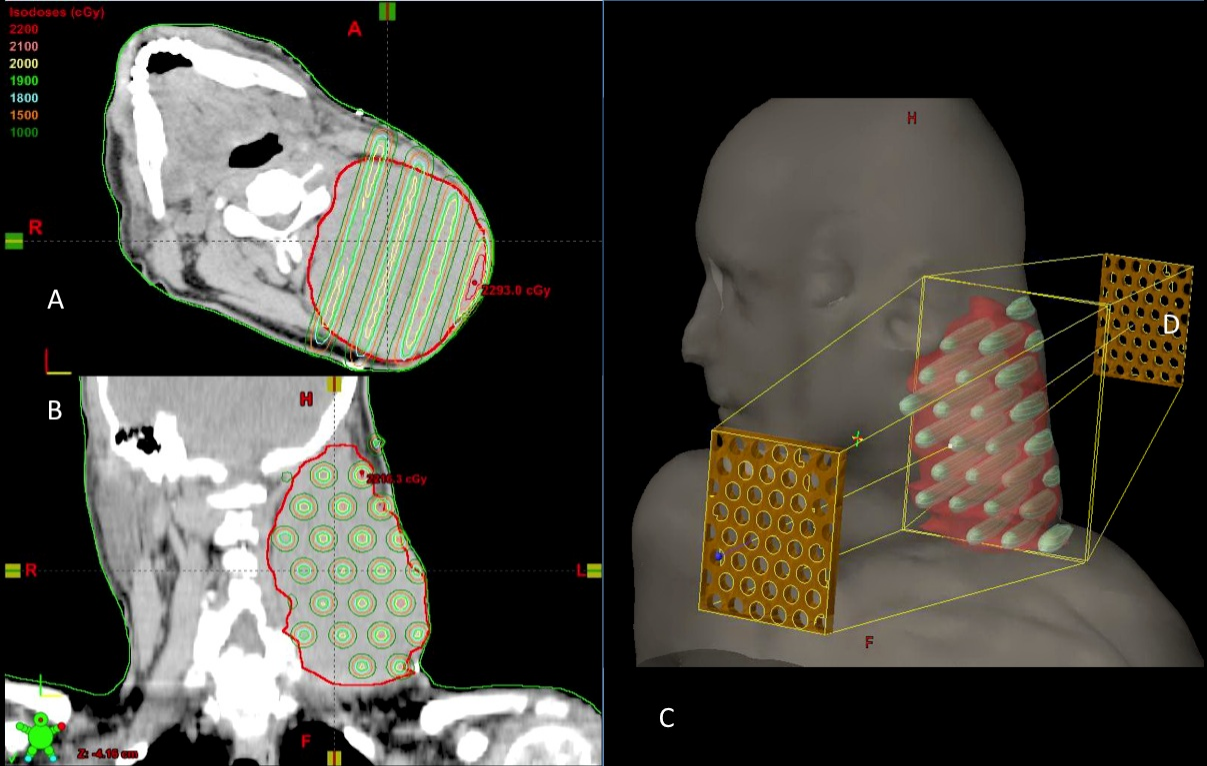
Radiation Dosimetry with GRID The initial tumor (red line) treated by GRID radiation of 20 Gy (yellow lines) in the A) axial, B) coronal, and C) 3-dimensional view with GRID blocks shown.

The tumor volume was 650 cm^3^ (10.7 cm equivalent sphere diameter) prior to treatment. He received 20 Gy in a single fraction using parallel opposed spatially fractionated GRID radiation therapy (SFGRT) to the left neck through a commercially available, specialized brass block (dot Decimal Inc., Sanford, FL). The GRID block treated the large tumor with high-dose beamlets spatially arranged in a 50:50 open-to-closed ratio (Figure 2).

This was followed by 50 Gy in 25 fractions of conventional radiation to the left neck completed by October 2014. The radiation was given with concurrent pembrolizumab. He had no side effects from the combination treatment.

**Figure 3:**
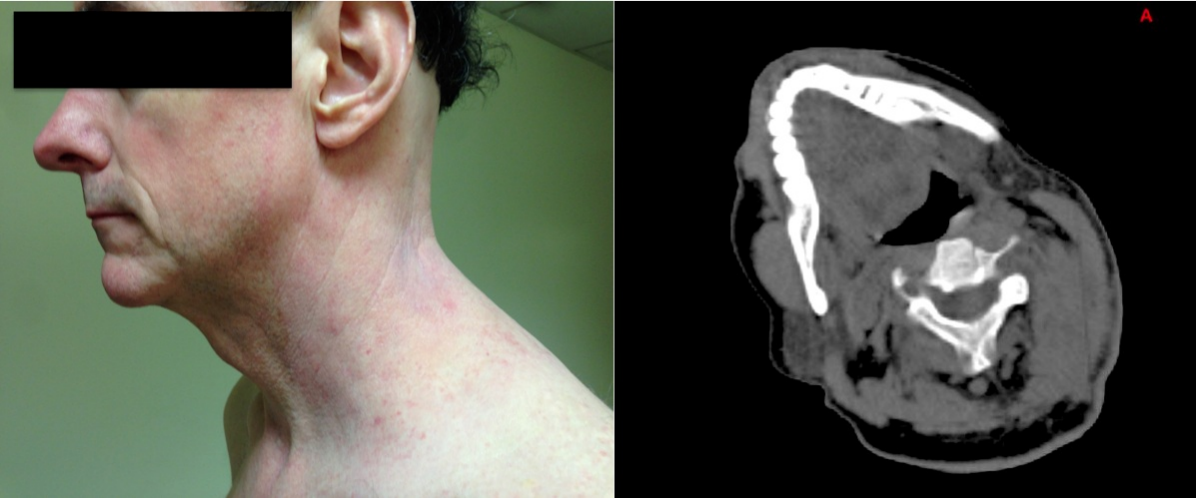
Tumor Response to Treatment The tumor is completely gone 5 months later clinically and on CT scan.

Two months after the treatment to the neck, the tumor mass had regressed over 75% by tumor volume, and clinically, he had a full range of neck motion and no adverse side -effects. By five months, there was complete resolution of the neck mass on CT scan (Figure 3).

The pembrolizumab was continued for a total of 18 cycles from June 2014 to June 2015. The retroperitoneal disease remained stable for six months and then started to progress. There has been no recurrence of tumor in the neck over the last 12 months from radiation.

## Discussion

We present a locally advanced melanoma tumor that initially responded to ipilimumab, but then progressed and became refractory to multiple systemic, immunological agents, including ipilimumab, high-dose IL-2, and pembrolizumab. We then introduced high-dose radiation along with the anti-PD-1 agent, pembrolizumab, and the patient had a rapid, complete response to treatment in the neck with minimal side effects.

The patient’s tumor had previously progressed on five cycles of pembrolizumab alone prior to radiation. Conventional radiation of 50 Gy alone would not generate the dramatic, rapid response seen here, either. Our case is important because it suggests that high-dose radiation can be used as an immunological primer for re-introducing sensitivity to biological agents. Formenti, et al. has suggested that the local radiation on the patient’s tissue acts as an in-situ vaccine [2]. Radiation targeted to the intact primary tumor can release radiation-specific antigens and induce attracting chemokines to activated T-cells, thus engaging the patient’s innate immune response against the tumor [3-4]. If the tumor-specific immune response is strong enough, it can also enable systemic regression in areas outside of the local radiation field, known as the abscopal effect [4].

Spatially fractionated GRID radiation therapy (SFGRT) is an ideal method for delivering a high dose of priming radiation to large-sized tumors with minimal side effects. It has been used with clinical success in a variety of radio-resistant tumors like melanoma and sarcoma [5]. The GRID block is made of 1.43 cm diameter circles spaced 2.11 cm apart (center-to-center) at 60-degree angles that allow delivery of multiple, very high-dose radiation beamlets to the tumor while also sparing considerable skin in the blocked areas. In a sense, it is similar to stereotactic body radiation therapy (SBRT), given its high dose per fraction, and it is like “non-invasive” interstitial brachytherapy, given its spatial dose delivery over a large area.

In short, the synergism of immunotherapy and local radiation dramatically upregulates the host’s immune response, even in tumors that were previously refractory to immune therapy. We have treated other locally advanced and refractory patients using this paradigm with similar success. Randomized trials are ongoing combining radiation with checkpoint blockade.

## Conclusions

Spatially fractionated GRID radiation therapy (SFGRT) delivers a high dose of radiation to large-sized tumors with minimal side effects. When combined with immunotherapy like the anti-PD-1 monoclonal antibody, pembrolizumab (Keytruda), it can prime the patient's refractory tumor to provide a new and dramatic synergistic immune response. Randomized trials are ongoing combining radiation with checkpoint blockade.
